# Factors Related to Self-Care in Heart Failure Patients According to the Middle-Range Theory of Self-Care of Chronic Illness: a Literature Update

**DOI:** 10.1007/s11897-017-0324-1

**Published:** 2017-02-17

**Authors:** Tiny Jaarsma, Jan Cameron, Barbara Riegel, Anna Stromberg

**Affiliations:** 1 Division of Nursing, Department of Social and Welfare Studies, Linköping University, campus Norrköping, s-581 83 Linköping, Sweden; 2 Mary Mackillop Institute, Australian Catholic University, Melbourne, Australia; 3 Department of Medicine, School of Clinical Sciences at Monash Health, Monash University, Clayton, Australia; 4 Australian Centre for Heart Health, Royal Melbourne Hospital, Parkville, Australia; 5 University of Pennsylvania School of Nursing, Philadelphia, PA USA; 6 Division of Nursing Science, Department of Medical and Health Sciences and Department of Cardiology, Linköping University, Linköping, Sweden; 7 Program in Nursing Science, University of California Irvine, Irvine, CA USA

**Keywords:** Self-care, Heart failure, Self-care maintenance, Self-care monitoring, Self-care management

## Abstract

**Purpose of the Review:**

As described in the theory of self-care in chronic illness, there is a wide range of factors that can influence self-care behavior. The purpose of this paper is to summarize the recent heart failure literature on these related factors in order to provide an overview on which factors might be suitable to be considered to make self-care interventions more successful.

**Recent Findings:**

Recent studies in heart failure patients confirm that factors described in the theory of self-care of chronic illness are relevant for heart failure patients.

**Summary:**

Experiences and skills, motivation, habits, cultural beliefs and values, functional and cognitive abilities, confidence, and support and access to care are all important to consider when developing or improving interventions for patients with heart failure and their families. Additional personal and contextual factors that might influence self-care need to be explored and included in future studies and theory development efforts.

## Introduction

Self-care is essential in the long-term management of chronic illnesses such as heart failure (HF). Self-care is defined as a process of maintaining health through health promoting practices and managing illness; self-care is performed in both healthy and ill states [1••]. Self-care can be seen as an overarching concept built from the three key concepts of self-care maintenance (e.g., taking medication as prescribed), self-care monitoring (e.g., regular weighing), and self-care management (e.g., change diuretic dose in response to symptoms). For patients with HF, it might be necessary to regulate and adapt self-care during the course of the illness, for example in times of deterioration, if co-morbidities occur, or in case of specific advanced treatment [1••]. Self-care has proven to be an important influence on both medical- and person-centered outcomes in patients with HF. Those who report more effective self-care have better quality of life, lower mortality and readmission rates than those who report poor self-care [2–4]. However, despite the obvious relationship of good self-care with positive health outcomes, many patients find it difficult to follow self-care advice. This lack of adherence might be related to the complexity of self-care, lack of perceived need for self-care, the long-term character of the behavioral changes needed, or a lack of motivation, to mention a few.

**Fig. 1:**
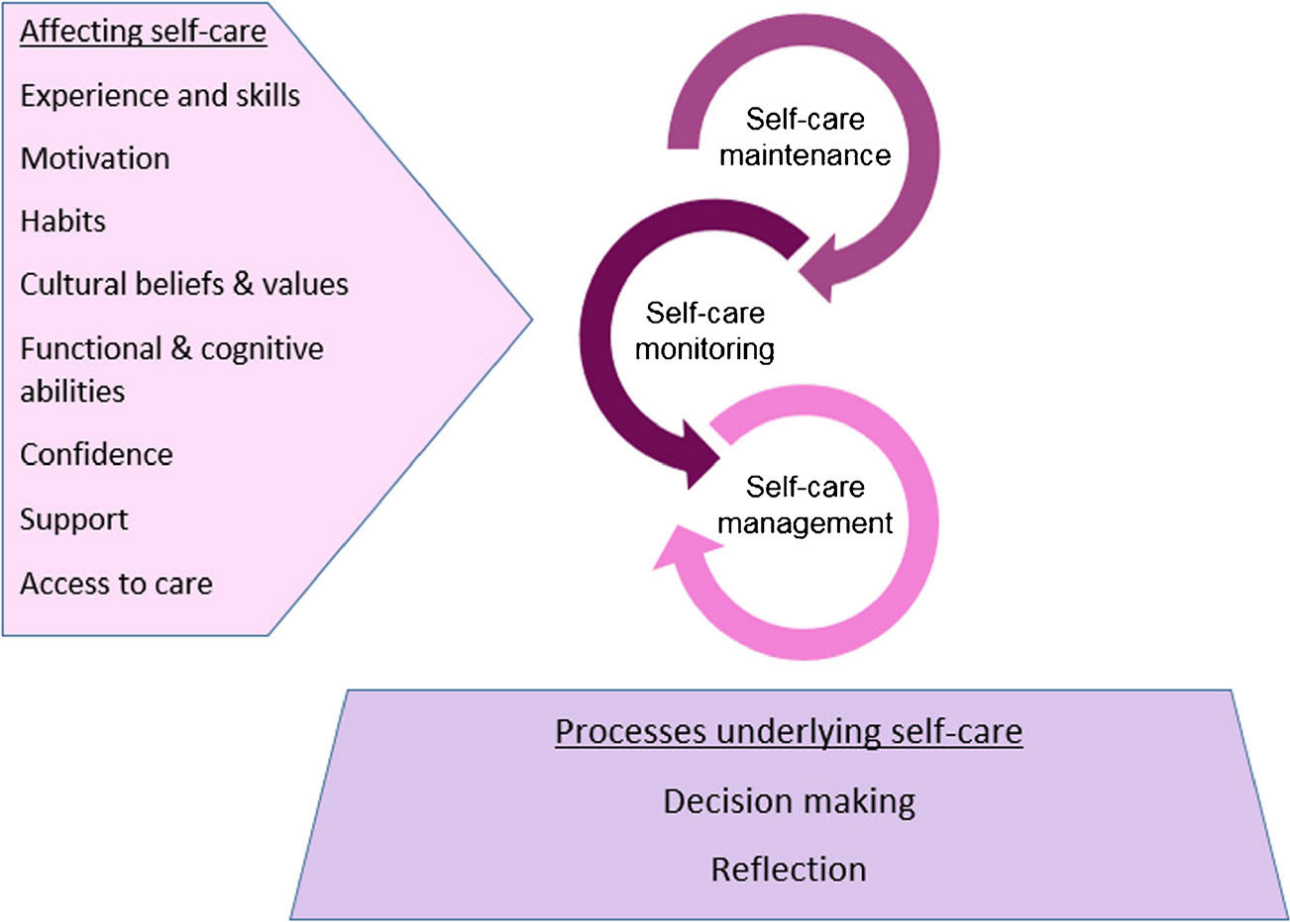
Factors affecting self-care and processes underlying self-care according to the middle-range theory of self-care of chronic illness

One challenge faced by clinicians is understanding the complex process of self-care and to develop appropriate, theory-driven interventions that support patients and their caregivers to maintain their health and manage their chronic illness. The middle-range theory of self-care of chronic illness [1••] and the situation-specific theory of heart failure self-care [5••] can assist clinicians in their assessment of patients with HF and in identifying individual factors that hinder their engagement in self-care. These theories also provide structure for researchers to test interventions directed at improving self-care that can be transferred into practice. Although several studies reported that patients with HF can change their self-care behaviors, the optimal self-care intervention to improve outcomes is not clear. A recent individual patient data meta-analysis of 20 randomized trials (5624 patients) evaluating self-care interventions in HF patients described that not all self-care interventions are effective [6••], Interventions that were of longer duration reduced mortality risk, risk of HF-related hospitalization, and HF-related hospitalization at 6 months. Although results were not consistent across outcomes, interventions with standardized training of interventionists, peer contact, log keeping, or goal-setting skills appeared *less* effective than interventions without these characteristics [6••]. The purpose of this paper is to summarize the recent HF literature on factors related to self-care behavior in order to provide an overview on which factors might be suitable to be considered to make future self-care interventions more successful. In Fig. 1, major factors identified as barriers and facilitators to self-care in the middle-range theory of self-care of chronic illness are presented. The current review examines the recent HF self-care literature in which these factors were studied.

## Experience and Skills

In a previously published middle range theory, we have stated that there are several factors influencing decisions about self-care, including knowledge, experience, and skills [1••]. Although several self-care interventions aim to increase knowledge of patients, we have come to recognize that knowledge is necessary, but insufficient to change self-care behavior [7]. In addition to acquiring knowledge, patients need to have the skill to plan, set goals, and make decisions. Experience is a powerful contributor to the development of skills in self-care. To gain sufficient experience and skill, patients need to understand the advice and instructions related to their self-care behavior and understand health information. In several recent studies, it has been confirmed that health literacy is independently associated with self-care behavior as found in studies in different populations and subpopulations [8, 9].

A recent review of 33 studies of HF disease management programs found that effective interventions were those that promoted understanding of the nature and complexities of HF and its self-care [10••]. Interventions that emphasized or reinforced the many and complex links between symptoms and HF self-care tasks were perceived to be particularly valuable.

Patients also need the ability to use information and apply it in a context, in other words, they need skills. Skills for HF self-care are described as evolving over time and with practice as patients learn how to make self-care practices fit into their daily lives and as they gain experience in successfully managing symptoms [7]. Different skills need to be targeted, such as tactical and situational skills, to improve different components of self-care [11].

## Motivation

Motivation is the force driving humans to achieve their goals [1••]. In a recent study, maintaining autonomy was the highest goal in the hierarchy of personal goals of patients with HF, followed by physical well-being, maintaining social relationships and symptom relief [12].

Motivations can be both intrinsic and extrinsic and both play a role in self-care behaviors. For example, in self-care behavior related to exercise, it was found that both intrinsic and extrinsic motivations were important but the intrinsic motivations (engaging in an activity for pleasure and inherent satisfaction) were more often expressed as motivators in patients with HF than extrinsic motivations (engaging in an activity because it was recommended by others) [13]. If patients see clear benefits, they may be more motivated. Early benefits to performing self-care are found to include reducing symptoms and improving quality of life. Later benefits relate more to promoting health [14]. Patients can be motivated for self-care by values related to personal feelings (self-direction, pleasure, and being healthy) or related to their life circumstances (maintaining a healthy lifestyle and financial balance). Patients are also motived by values that are socially based: benefits received from society (social recognition and socialization) and social obligations (responsibility, observing traditions, and obedience) [15••].

## Habits

Habits or daily routines are powerful influences on self-care [1••]. Learning from past experiences can enable patients and caregivers to apply self-care strategies in daily activities. Some patients however have problems in forming habits or integrating self-care in their daily lives. Even if patients get self-care advice from health professionals they may not integrate this knowledge into daily life or make it into a habit. Attempts to manage HF might remain based on how patients ‘feel’ rather than clinical indicators of worsening symptoms [16]. To integrate self-care in daily life, education should include strategies that promote self-efficacy, learning and application of the recommendations to daily life [16].

Behavioral economics is an approach that is being used successfully to modify habits. Behavioral economics is a relatively new field that harnesses predictable patterns of behavior like habits, which often lead people to make choices that are not in their best interest [17]. Studies based on behavioral economics reward existing behavioral patterns to facilitate better health. For example, intervention programs encourage patients to use self-control, which few of us possess. Conversely, programs based on behavioral economics offer patients small and frequent payments for behavior that would benefit them, such as medication adherence. These types of intervention can be more effective because they reward behavior rather than control it [18].

## Cultural Beliefs and Values

Cultural beliefs and values can affect self-care behavior because of different availability of recourses or differences in priority setting between the various self-care behaviors [19•, 20•]. Self-care theory has been criticized for being too focused on Western culture [21], but in spite of this criticism, self-care is studied in many widely varying cultures from around the world. When comparing self-care behaviors in HF patients from 15 different countries, we found that several differences existed across and within cultures, countries, and continents. Some self-care behaviors might be easily implemented, while others might not be implemented in certain cultures [19•, 20•]. For example, in some cultures, the concept of karma strongly influences people’s beliefs and their way of thinking and living. Patients with HF might incorporate their religious and cultural beliefs to rationalize HF symptoms experienced and help them come to terms in living with such a disabling chronic condition [22]. Another example is that in certain cultures patients will leave decision-making responsibility to their healthcare providers, who are held in high regard for their positions of responsibility [22, 23]. In these situations, patients may not feel responsible or adequate to make decisions about self-care. Although the majority of studies is reported from westernized countries, an increasing number of studies examine culturally specific education programs such as those recently reported from Thailand, Japan, and Lebanon [24–26].

Elements of particular cultural interest that can be incorporated to improve future self-care interventions include addressing eating habits, social connection, and collectivism. Even within countries, different cultural groups and minorities have different self-care challenges. Low income ethnic minority patients have reported difficulty in adherence to a prescribed diet due to conflict with cultural food preferences and family roles [27]. A Chinese study found that cultural factors influencing dietary and fluid restriction behavior included the values placed on health and illness, customary way of life, preference for folk care and the Chinese healthcare system, and factors related to kinship and social ties, religion, economics, and education [28].

Values of patients also greatly influence heart failure self-care. In a recently published mixed-methods systematic review, it was found that patients make their decisions about self-care based on the values they prioritize and those that are blocked. Patients were motivated for self-care by values related to personal feelings (self-direction, pleasure, and being healthy) or related to individuals’ life circumstances (maintaining a healthy lifestyle and financial balance). Patients were also motivated by values that are socially based, some are related to benefits received from society (social recognition and socialization) and social obligations (responsibility, observing traditions, and obedience) [15••]. Further, treatment beliefs about for example the necessity of medications or the belief in the illness having serious consequences influences self-care have been shown to be significant predictors of self-care [29].

## Functional and Cognitive Abilities

To engage in self-care behavior, a patient needs to have certain functional and cognitive abilities that enable him/her to stand on a scale, make choices for healthy food, call a health care provider, etc. Other functional abilities that can restrict patients from engaging in good self-care include problems with hearing, vision, manual dexterity or balance [1••, 30]. In addition, general and exertional fatigue are significantly associated with poor HF self-care and poor consulting behavior over time, independent of sleep and mood problems, and other clinical factors [31••]. Furthermore, cognitive impairment, anxiety, and depression can decrease the ability and interest in performing self-care [31••].

Cognitive impairment is a major issue in patients with HF, with prevalence rates reported between 25 [32] to 80% [33], depending on how cognition is assessed and classified. When memory, attention, problem-solving, and psychomotor speed are even slightly impaired, it can negatively impact on patient engagement in HF self-care [34–38]. In a recent narrative synthesis, growing evidence was found regarding the association between mild cognitive impairment and low self-care behavior in HF patients. Nine of ten studies reported significant positive associations between mild cognitive impairment and HF self-care, either specifically in relation to medication adherence or more generic measures of self-care behaviors [34].

Taking account of some of the factors that influence self-care such as age, gender, education, income, and social support, it is likely that cognitive impairment interacts and combines in different ways with these factors to influence overall patterns of self-care. For instance, Vellone et al. identified four patterns of HF self-care; each cluster was explained by specific sociodemographic and clinical characteristics [4]. In particular, persons with inconsistent self-care and high consulting behaviors were characterized as mostly female, with lower formal education, poor cognitive impairment, poor physical and mental quality of life. Although cognitive deficits are not associated with adherence to daily weighing, adults with cognitive deficits may be at an increased risk for experiencing a clinically significant weight gain and not perceiving symptoms [39]. A recent study demonstrated the importance of memory in the specific self-care behavior of medication adherence [40].

Preliminary work suggests that cognitive training may make a small improvement in cognitive function among patients with HF [41, 42]. Cognitive training also had positive effects on engagement in HF self-care and functional outcomes such as instrumental activities of daily living (e.g., medication taking, grocery shopping).

## Confidence

Self-care confidence is an important factor influencing HF self-care and interventions addressing confidence should be considered as a way to improve self-care in this population [43]. In fact, one study found that self-care confidence was more important than cognition in predicting HF self-care [43]. In studies with HF caregivers, confidence also has been shown to be important. Vellone et al. [44] found that caregiver confidence in the ability to contribute to the HF patient’s self-care explained a significant amount of variance in the caregiver’s contributions to patient self-care. Those caregivers with more confidence were more helpful to HF patients performing self-care. In an interesting recent dyadic study of both HF patients and their caregivers, patient and caregiver levels of confidence were significantly associated with greater patient-reported relationship quality and better caregiver mental health [45]. Patient confidence in self-care was significantly associated with poor caregiver physical health. Caregiver confidence to contribute to self-care was significantly associated with poor emotional quality of life in patients.

In a study on specific self-care behaviors in patients with chronic HF, Kessing and colleagues described that besides depression and mental well-being, self-efficacy was associated better self-care. These associations were predominantly observed with self-reported indices of self-care and not objective measures [31••].

## Support

Family and friends often play a critical role in supporting patient engagement in self-care [1••]. In a qualitative systematic review including 45 studies on self-care in HF patients, it was found that of the six main types of contextual factors influencing HF self-care were caregivers: social networks and social support, place, finances and financial capacity, work and occupation, and HF support groups and programs [46••]. Caregivers make a vital contribution to patient’s self-care [1••] and in their day to day lives many patients with HF depend on the support of their families or social network [47]. In addition to practical and motivation support, social support may influence depressive symptoms and self-care behaviors, whereas social problem-solving may impact self-care behaviors [48]. However, at the same time, carers’ mood states also can have a detrimental effect on patient self-care maintenance and management behaviors [49], as described above.

How support for patients with HF can be maximized is still the subject of discussion and study [48]. There are different levels of partner participation, with most partners being satisfied with their participation in care, but some partners fear future demands [50]. In cultures were collectivism prevails, family members often live with the patient and are available to provide all aspects of support. Hence, the family member is considered a crucial partner and should be included in all aspects of a self-care intervention [25].

## Access to Care

Although most self-care is performed by patients and family members at home, self-care is often influenced to some degree by providers after accessing the health care system to obtain care. In a qualitative study, both patients and their partners emphasized the importance of having guidance and receive education on self-care by heath care professionals through the different phases of the illness trajectory. Regular outpatient clinic visits to a HF nurse and easy access to care were highlighted by the dyads as being very important to support heart failure self-care [51]. Rural patients often have issues with access and a growing body of research addresses self-care in rural patients. In one study [52], HF patient activation level was low (e.g., taking no action to manage their HF) and low patient activation was associated with inadequate HF knowledge, low confidence, and poor self-care management after hospital discharge. In another study of rural HF patients, Caldwell and colleagues found that a simplified education program designed for use in resource scarce settings improved knowledge and patient-reported self-care behaviors [53]. Programs such as this should seek to harness the main mechanisms through which interventions actually work to improve HF self-care and outcomes, rather than simply replicating components from other programs. The most promising mechanisms to harness are associated with increased patient understanding and self-efficacy, involvement of other caregivers and health professionals, improving psychosocial well-being and technology use [10••].

## Conclusion

This review summarized the recent HF literature describing the factors that might be related to self-care as mentioned in the theory of self-care of chronic illness. The factors mentioned in this theory include experience and skills, motivation, habits, cultural beliefs and values, functional abilities and cognitive abilities, confidence, support and access to care, and recent literature confirms that these factors are relevant in heart failure self-care. A considerable number of studies have addressed the relationship of cognition and heart failure while only a few studies focused specifically on motivation and building habits and daily routines.

In addition to these factors, several contextual factors or processes underlying self-care behavior have to be considered. For example, a recent review has summarized decision-making in relation to heart failure care. There were 12 studies addressing this topic, but the majority of the studies focused on decisions about end-of life care; while only one third focused on decisions about self-care behaviors. Decision making as a concept was often unclear or poorly defined and multiple limitations in study design and methodological rigor limited definitive conclusions about heart failure decision making in relation to self-care.

HF self-care might be influenced by a great variety of factors that may not be addressed currently in HF self-care programs. It is known that HF patients have difficulty with self-care, and the influence of cognitive function needs to be considered when providing professional support. To optimally develop future self-care interventions, current knowledge on factors influencing self-care as summarized in this review should be taken into account. Further, insights related to personal and contextual factors that might influence self-care need to be explored and included in future studies and theory development.
